# Project to Review the Medical Appraisal Policy in Tees Esk and Wear Valley (TEWV) NHS Trust and Implementation of the Outcomes

**DOI:** 10.1192/bjo.2024.369

**Published:** 2024-08-01

**Authors:** Sachin Gandotra

**Affiliations:** Tees Esk and Wear Valley NHS Trust, Middlesbrough, United Kingdom

## Abstract

**Aims:**

The project aimed to review the Trust Medical Appraisal policy and offer a platform to update the Trust policy locally and align it to a National recommendation in the Medical Appraisal Guide besides gathering consensus for change for other relevant issues to the Trust.

**Methods:**

The project was undertaken as a part of the ‘Leadership and management fellowship Scheme’ sponsored by the Tees Esk and Wear Valley NHS Foundation Trust and conducted in collaboration with the Royal College of Psychiatrists, UK and Faculty of Leadership and Management, UK 2022–23 with data collection lasting from January till August 2023. The methodology consisted of drafting a document comparing the information from the review of the existing Trust medical appraisal policy and the guidance in the Medical Appraisal guide, drafting a questionnaire which covered the complex issues in the appraisal process and where the Trust medical appraisal policy was identified as having gaps which required further opinions to be generated for a possible revision to the policy, and gathering consensus opinions from focus group discussions for different groups of staff which included appraisers who are not managers, consultants who are not appraisers, medical managers who are not appraisers, consultants who are appraisers and SAS doctors who are not appraisers. The focus groups were conducted virtually as well as face to face groups and consensus opinions were then synthesised with information available from the guidelines to draft recommendations. The recommendations were then presented to the senior managers in the Trust appraisal process to seek feedback and approval.

**Results:**

The main recommendations that followed from the review were: to promote supportive and developmental nature of the appraisal process by making the process less document intensive by modifying appraisal portfolio and appraisal sections, educating staff on not duplicating information, promote verbal reflection, and modifying corporate supporting information section to reduce burden on doctors; maintaining 3 year appraiser turnover; avoiding line manager to be the appraiser of the appraisee; not sending appraisal summary to the line manager and considering how to facilitate communication and input of the line manager to the revalidation decision; clarifying requirements of supporting information for appraisal of particular group of doctors (Trust doctors, International Medical Graduates (IMG), academics, and on zero hour contracts); expand corporate supporting information to include General Medical Council (GMC)/Trust disciplinary and low level concerns; to promote wellbeing discussion by adding prompt for doctor to comment on their wellbeing; adding a wellbeing statement to the appraisal template and finally to add trainer accreditation statement to the appraisal template to facilitate reporting of trainer accreditation. Most of the recommendations were accepted by the Trust except one on expanding the corporate supporting information for doctors and addition of a wellbeing template in appraisal section.

**Conclusion:**

The project served as a significant leadership experience in my training role to undertake a project driving a Trust-wide change in medical appraisal policy based on participative leadership, generating consensus and developing a phased action plan towards implementation.

